# Using Big Data for Clinical Decision Making

**DOI:** 10.1093/geroni/igaa057.563

**Published:** 2020-12-16

**Authors:** Diana Woods, Maria Yefimova

**Affiliations:** 1 Azusa Pacific University, Marina del Rey, California, United States; 2 VA Palo Alto Healthcare System, Redwood City, California, United States

## Abstract

The current workforce is ill prepared for the rise in Americans 65 and older from 46.3 million in 2010 to 98.2 million by 2050, a national increase of 112.2 % accompanied by increasing chronic conditions. The increase in older Americans, the prevalence of those with dementia, accompanied by behavioral symptoms of dementia (BSD) is increasing. Innovative technology may alert health providers to early signs of decline in frail older adults with multiple chronic conditions. Remote monitoring in the home and community living spaces can address complex care needs for older adults. Monitoring may identify and predict deviations in a person’s daily routine that herald a change in a chronic condition. We present two examples that can potentially assist in clinical decision making. The first exemplar used 24/7 sensor data to identify changes, potentially clinically significant, such that early intervention may prevent hospitalizations; the second exemplar presents the use of pattern recognition software (THEME TM) for temporal pattern analysis, to identify and quantify behavior patterns with regard to intensity, frequency and complexity, such that interventions may be individually tailored and timed. Clinical researchers and technology developers need to collaborate early in the process to consider the sources and frequency of clinical measures for meaningful predictions. One major challenge lies in the interpretation of the vast amounts of within individual data. Our insights strive to improve future interdisciplinary development of monitoring systems to support aging in place and support clinical decisions for timely and effective care for frail older adults.

